# Analysis of experimental conditions for alkali activation of mining tailings based on literature data

**DOI:** 10.1007/s11356-026-37892-9

**Published:** 2026-06-03

**Authors:** Lucimara Bragagnolo, Pedro Domingos Marques Prietto, Eduardo Pavan Korf

**Affiliations:** 1 Graduate Program in Civil and Environmental Engineering, University of Passo Fundo (UPF), CEP, Campus I, Km 171, BR 285, Passo Fundo, Rio Grande Do Sul 99001-970 Brazil; 2 Graduate Program in Environmental Science and Technology, PPGCTA, Federal University of Fronteira Sul (UFFS), Campus Erechim, Km 72, RS 135, Erechim, Rio Grande Do Sul 99.700-000 Brazil

**Keywords:** Mining waste, Activator solution, Data compilation, Mechanical strength

## Abstract

The growing focus on utilizing mining waste in construction, driven by environmental concerns, underscores its potential significance as global annual tailing production surpasses 7 billion tons. Alkaline activation of mining waste emerges as a promising approach, providing a sustainable alternative for creating value-added materials. Despite numerous studies on this subject, finding optimal ranges of values for process variables remains challenging. In this context, an evaluation was sought based on experimental conditions from the literature data to understand the relationships between experimental conditions and the mechanical response of alkali-activated products with mining rejects. The analysis reveals that the type of precursor significantly influences mechanical strength. Also, combinations of tailings and co-precursors demonstrate superior performance compared to using 100% tailings. NaOH and sodium silicate prove to be predominant activators, and the addition of co-precursors enhances mechanical strength. The study suggests the need for more flexible conditions, including alternative activators and co-precursors, to reduce environmental and financial costs in real-world applications.

## Introduction

The utilization of mining tailings in the construction industry has been gaining significant attention due to its potential to address environmental and sustainability concerns. Moreover, the mining industry generates substantial amounts of tailings (MTs), with an estimated total annual quantity exceeding 7 billion tons globally (Perumal et al. 2021) and by 2025, an expected amount of 19 billion tons (Araujo et al. 2022).

In this context, the effort to repurpose mining waste can play a vital role in decreasing the volume of waste that needs to be discarded. The concept of circular economy, the ability to be reused, the reuse process, and the act of reutilization have been recognized as emerging solutions with the capacity to drive multidimensional facets of sustainability within the mining and metal extraction sectors (Araujo et al. 2022).

The alkali activation of tailings has emerged as a promising approach to transform these mining residues into value-added construction materials. Alkali-activated materials have been identified as a sustainable alternative for the global construction materials industry (Provis 2018). The potential to use mining waste in alkali-activated cements and cementitious composites has been the subject of considerable research interest (Kinnunen et al. 2018). Studies have shown that mining waste contains essential elements such as silicon, aluminum, and calcium, crucial for alkali activation, indicating their suitability for this process (Aseniero et al. 2019). For example, Wang et al. (2019) investigated the feasibility of using iron ore tailings to produce geopolymer bricks, using sodium silicate solution as the activator. Also, Levandoski et al. (2023) demonstrated the successful stabilization of iron ore tailings using an alkali-activated binder, leading to improved mechanical behavior and more compact structures with longer curing times. Another study used garnet tailings as a precursor for geopolymer synthesis, with metakaolin as the main precursor and sodium silicate as the alkaline activation agent (Manjarrez and Zhang 2018). Copper mine tailings have also been explored as an alternative material for road base construction through alkali activation, with the maximum dry unit weight and unconfined compressive strength being influenced by the molarity of the NaOH solution (Kuranchie et al. 2016). Additionally, gold mine tailings rich in Si and Al oxides were activated by an alkaline hydrothermal method to produce a one-part alkali-activated material, resulting in a significant increase in compressive strength compared to the control sample (Zhang et al. 2011).

Although several studies have evaluated different experimental conditions to produce alkali-activated materials with mining waste, finding optimal ranges of values for the variables remains a challenge (Longos et al. 2020; Zhao et al. 2021). This is due to various factors: the composition of mining tailings can vary significantly, influencing the effectiveness of the alkaline activation process; the optimization of process parameters, such as the concentration of the alkaline solution and curing conditions, requires careful experimentation and analysis; the safety and environmental impact of the process must be considered, as it involves the use of potentially hazardous chemicals; and the economic feasibility of the process must also be taken into account (Gao et al. 2023).

Understanding the optimal conditions for the alkaline activation of mining waste is crucial to maximize the effectiveness of this process. Additionally, analyzing data from previous studies can provide valuable insights into trends and patterns in the alkaline activation of mining waste. In this context, one approach to address these issues is to examine patterns in existing experimental data.

With this approach in mind, we sought to evaluate, based on the existing literature on tailings used as precursors in alkaline activation, the relationships between the experimental conditions assessed by the studies. So far, no other studies have been identified that have conducted this type of analysis in this specific context. It is important to note that this study does not aim to provide a comprehensive review of mining waste in alkaline activation. Instead, it focuses on analyzing experimental characteristics related to product strength, compiling data from various studies.

This approach can offer a more comprehensive understanding of how variables can influence the outcome, considering different types of precursors, alkaline activators, and combinations—aspects that a single experimental study cannot cover due to time and cost limitations. Additionally, the study may provide insights into existing gaps in experimental conditions.

## Methodology

The methodological process involved a series of steps, which will be described in the following sections: (a) collection of existing literature on the alkaline activation of mining tailings, (b) application of criteria and selection of relevant articles, (c) extraction of information from the selected studies, (d) generation of tabulated data, (e) evaluation and generation of results.

### Literature search

Documents pertinent to the study’s scope were gathered from the Scopus and Web of Science databases. These databases are globally recognized and utilized across a wide range of research fields, with impact assessments based on citation counts and other bibliometric indices.

**Fig. 1:**
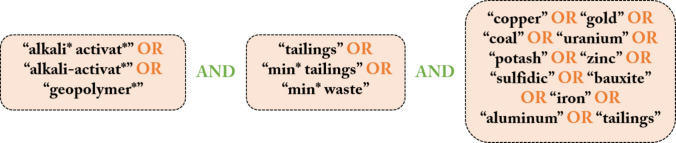
Boolean operators and keyword combinations applied for the search in each database

Two distinct searches were performed on each database using the same keyword set, combined using the Boolean operators “AND” and “OR”. The “AND” operator ensures that the selected keywords appear together in the publication, while the “OR” operator allows for the combination of one keyword or another. Documents were selected if they contained the keywords in the title, abstract, or keyword sections, using the “TITLE-ABS-KEY” operator for Scopus and “TS” for Web of Science. Quotation marks were employed to treat a combination of keywords as a single search term. The Boolean operators and the combinations that were used are illustrated in Fig. 1. The use of star marks (*) makes it possible to identify keyword variations, as shown in Table 1. In addition to the primary keywords like “alkali activation,” “geopolymers,” “tailings,” and “mine waste,” a variety of tailing types from diverse origins were specified. This was done to capture the broadest possible range of titles relevant to the study’s scope. It is also crucial to note that this study focused on the combined use of tailings and alkali activation. Other by-products of the mining process, such as gangue and slag, were not considered.

**Table 1:** Keywords and their possible variations

Keyword	Possible variations
alkali* activat* or alkali-activat*	Alkali activation; alkaline activation; alkali activated; alkaline activated; alkali-activation; alkali-activated
geopolymer*	Geopolymer; geopolymers; geopolymerization; geopolymerisation
min* tailings	Mine tailings; mining tailings
min* waste	Mine waste; mining waste

### Studies selection

The keyword search depicted in Fig. 1 yielded 242 documents from Scopus and 193 from Web of Science, resulting in a total of 435 bibliographic entries spanning the period from 1991 to 2021. The data from both databases was consolidated, and duplicate entries (130 documents) were removed, leaving 305 references. To focus solely on documents within the proposed scope (alkali activation of mining tailings), an evaluation of titles and abstracts was conducted. This led to the removal of entries that separately addressed alkali activation or tailings. Documents that reported the use of gangue, fly ash, or slags were also excluded, as these mining by-products are not classified as tailings. The inclusion of documents beyond the intended scope was due to the use of the keyword “min* waste,” which covers a wider range of mining by-products. Despite this, the keyword was chosen to ensure the inclusion of documents that also classify tailings as mining waste. Following this initial filtering process, 178 bibliographic entries were selected. The year 2021 was established as the upper temporal boundary of the search, as the literature collection was conducted as part of a doctoral thesis with a predefined methodological protocol approved prior to data extraction. This boundary is an inherent characteristic of the study design and does not reflect a limitation introduced post hoc.

The second filtering stage involved removing review articles, conference papers, books, and book chapters, as well as studies lacking information considered essential for analysis, such as data on the chemical composition of precursors, activators, and strength analyses. Table 2 shows the remaining quantity of articles selected for the subsequent stages:

**Table 2:** Quantity of initial articles and documents removed for further analysis

Total initial articles for analysis	178
Review articles	20
Conference papers	25
Books and book chapters	2
Outside the scope or lacking sufficient information	68
Articles OK for analysis	63

From the remaining total, a table was created in Excel with the following extracted information:

- Author, year of publication, and title
- Type of mining tailings used in the study
- Co-precursors (if used)
- Methodological considerations
- Type of alkaline activator used
- Activator concentration
- Pre-treatments (if any)
- Particle size information
- Chemical composition
- Molding moisture
- Curing time
- Curing temperature
- Curing method
- Mineralogy
- Unconfined compressive strength (UCS)
- Durability tests conducted
- Specimen size
- Leaching tests conducted
- Other types of tests and assessments evaluated

### Data analysis

To evaluate the influence of experimental conditions, we carry out both difference analysis and correlation analysis. Understanding the relationship between variables and identifying existing patterns among them is of utmost value to assist in optimizing alkali activation processes using mining tailings as precursors, as well as providing important information for data-driven decision-making.

For this study, the mechanical response was assessed based on the results of unconfined compressive strength (UCS) tests. This dependent variable was defined after conducting a bibliometric review and data collection, where it was observed that most studies assessed the efficiency of alkali-activated systems based on this type of mechanical test (Bragagnolo et al. 2022). Using other responses would narrow the scope of the proposed analysis, as a very limited number of studies conducted any other type of mechanical evaluation.

Initially, univariate statistical methods were applied for a better understanding of the distribution and properties of each variable to be evaluated. To assess the influence of experimental conditions, both difference analysis and correlation analysis were performed. As the data did not follow a normal or lognormal distribution, it was inappropriate to use a *T*-test for difference analysis. Therefore, the Mann-Whitney *U* test was chosen instead.

All analyses and figures were developed using the Python language and libraries such as pandas, numpy, seaborn, and scipy.

## Results

Based on the information available and collected for each of the variables mentioned in the methodology (Sect. “Studies selection”), the main variables that were analyzed and discussed in the following sections are tailing type, type and concentration of activators, use of pre-treatment, the influence of co-precursor, temperature, curing time, and liquid-to-solid (L/S) ratio.

Furthermore, an initial correlation analysis was developed to determine the influence of experimental conditions. However, none of the selected conditions showed a strong correlation with the response variable UCS. This is likely an indication that the measured values from various studies are not highly consistent with each other. Therefore, the results that will be presented are based on distribution analyses and difference analyses using statistical tests, as mentioned in the methodology (Sect. “Data analysis”).

### Tailings

**Fig. 2:**
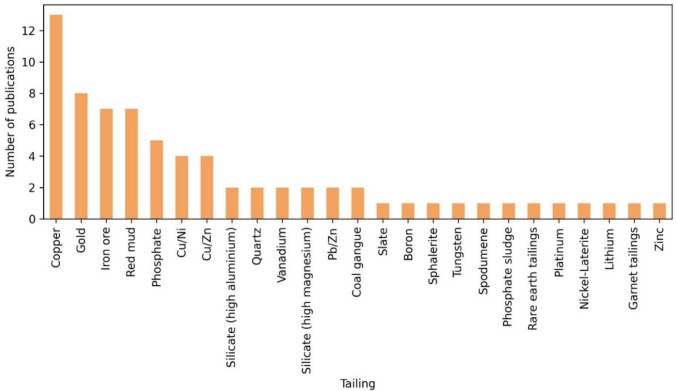
Mining tailings used in studies on the production of alkali-activated materials

In Fig. 2, it is possible to identify the mining tailings most used as precursors in the alkaline activation process in the evaluated studies. Copper tailings were the most studied, followed by gold, iron, and red mud tailings. These tailings exhibit distinct chemical characteristics that can influence their chemical reactivity and the performance of materials produced from them. It is important to understand the specificities of each to maximize their use as precursors in alkaline activation.

**Fig. 3:**
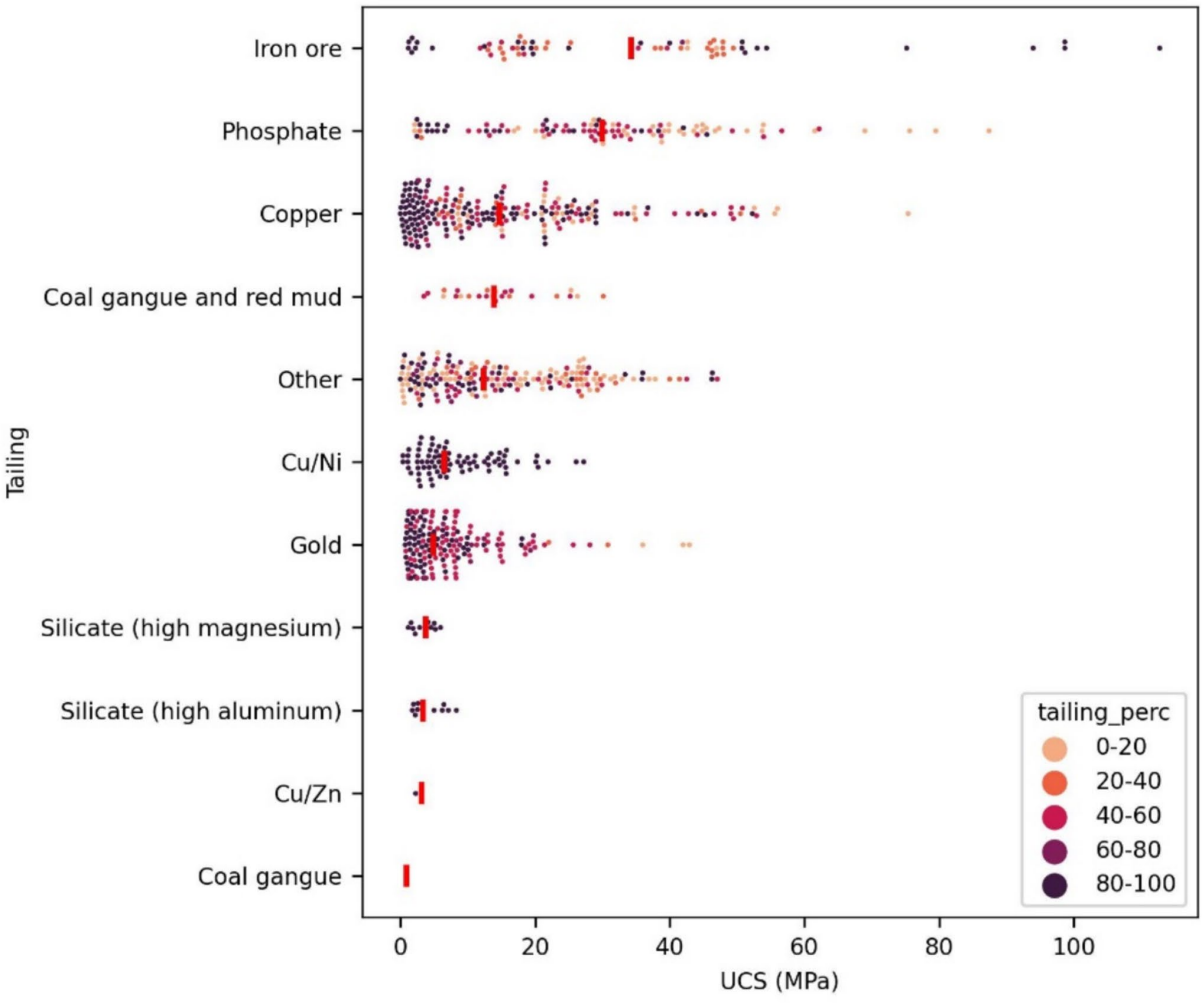
Distribution of UCS values for each type of mining tailings used in alkali activation processes. The colors represent the percentage of tailings used in the mixture with another co-precursor in each of the samples. The red bar represents the median

Figure 3 presents the distribution of unconfined compressive strength (UCS) values concerning different mining tailings used as precursors in alkali activation found in the literature. The red bars represent the median UCS value for each tailing. It is observable that the tailings showing the highest median UCS values were iron and phosphate tailings.

It is important to note that the UCS distributions shown in Fig. 3 should not be interpreted as a direct ranking of tailing types based on their intrinsic reactivity or mechanical potential. The strength values reported for each tailing type result from the simultaneous influence of multiple experimental variables, including activator type and concentration, co-precursor content, pre-treatment method, and curing conditions, which varied substantially across the compiled studies. This complexity is further supported by the findings presented in Sect. “Influence of co-precursor” (influence of pre-treatment), where the Mann-Whitney statistical test revealed no statistically significant difference in UCS between samples with and without pre-treatment for groups containing tailings (Fig. 11), indicating that no single variable can be considered in isolation. The purpose of Fig. 3 is therefore not to establish a hierarchy among tailing types, but to contextualize the range of UCS outcomes reported in the literature for each tailing and to identify which combinations of experimental conditions were associated with higher or lower performance within each group.

It should also be acknowledged that classifying tailings by type (e.g., copper tailings, iron tailings) does not fully capture the substantial compositional variability that exists within each category, as the SiO₂, Al₂O₃, and CaO contents of tailings from different deposits can vary considerably and directly affect their reactivity in alkali activation. While the chemical composition of precursors was extracted as part of the data collection (see Sect. “Studies selection”), a cluster analysis based on oxide ratios such as CaO/SiO₂ and Al₂O₃/SiO₂ was not performed in the present study. This was primarily because most compiled samples involved mixtures of tailings with co-precursors in varying proportions, meaning that the effective chemical composition of each system is a weighted combination of multiple components—a complexity that would make compositional clustering of the tailing fraction alone misleading. Additionally, the compositional data reported across studies were not sufficiently standardized (e.g., differences in whether values are reported for the tailing alone or the final blend, and variations in analytical conventions) to support a robust multivariate grouping. These represent important limitations of the present analysis and suggest that future work employing more controlled and compositionally documented datasets could significantly benefit from such approaches.

For studies using iron ore tailings (IOT), it was observed that the highest UCS values are strongly related to a low percentage of tailings in the mixture and higher concentrations of alkali activators. In a study by (Cao et al. 2013), high strengths were achieved with mixtures containing at least 50% calcined kaolin. The authors noted that as a higher percentage of tailings was added, UCS values decreased. For (do Carmo e Silva Defáveri et al. 2019), who used only IOT to develop the alkali-activated system, strengths above 50 MPa were observed with the use of relatively high concentrations of NaOH (8–12 M) and grinding for up to 3 h as a pre-treatment, with high curing temperatures (100 °C). Similar behaviors were observed in the study by (Kumar et al. 2017). Lower UCS ranges for IOT were associated with experiments involving mixtures with metakaolin and a combination of activators (NaOH with sodium silicate), along with the use of relatively lower curing temperatures (ambient temperature up to 60 °C) and without any pre-treatment (Obenaus-Emler et al. 2020).

Similar UCS behaviors are observed in experiments involving the use of phosphate as a precursor. Moukannaa et al. (2018) identified that adding phosphate sludge as a partial substitute for fly ash and metakaolin in alkali-activated compound production results in a reduction in mechanical properties. However, the highest strengths for 50/50 phosphate/fly ash and 50/50 phosphate/metakaolin mixtures were observed at the highest tested curing temperature (83 °C) and higher concentration of alkali activator (12.5 M). Other studies with phosphate show averages and maximum UCS values within the commonly observed range. Still, they remain above the overall calculated median (10.6 MPa) (Perumal et al. 2019; Niu et al. 2020; Wu et al. 2020; Tome et al. 2021).

Regarding copper, it is also generally observed that points above the median value are related to samples that used high concentrations of NaOH (~ 15 M) (Ahmari et al. 2012; Ahmari and Zhang 2013a; Manjarrez et al. 2019), often associated with some pre-treatment process (usually grinding) (Ren et al. 2015; Tian et al. 2020a) and high curing temperatures (above 90 °C) (Ahmari and Zhang 2013a, b; Ren et al. 2015), as well as mixtures with co-precursors (Ahmari et al. 2015; Manjarrez et al. 2019; Tian et al. 2020c, a). From Fig. 3, it is possible to identify that, for cases where some type of co-precursor was used in the mixture, the samples with higher resistance are those with a higher percentage of co-precursor than the tailings themselves (points with lighter colors). For example, Ahmari et al. (2015) obtained UCS above 70 MPa when only low-calcium slag was used (0% copper). For samples with 100% tailings, maximum values of 20 MPa were reached. Values in this range were also found in experiments with copper without pre-treatment, even using relatively high curing temperatures (90 °C) and high molarity of alkali activator (15 M) (Ahmari et al. 2012).

For the other types of tailings presented in Fig. 3, UCS values mostly concentrate below 20 MPa, represented by samples with no or a low percentage of mixture with another type of co-precursor, corroborating with the behavior of the results presented earlier.

Taken together, the results presented in Fig. 3 reveal a consistent cross-tailing pattern: regardless of the tailing type, samples achieving the highest UCS values tend to share a recurrent set of experimental conditions, namely the use of a co-precursor at proportions exceeding 40%, curing temperatures above 80 °C, and activator concentrations at the higher end of the studied range. This convergence suggests that the chemical nature of the tailing itself may be a secondary factor in determining the upper bound of mechanical performance, compared to the combined effect of process conditions. Conversely, the wide spread of low UCS values observed for all tailing types, including those with higher median values such as iron and phosphate, indicates that adverse combinations of these variables consistently undermine performance regardless of precursor origin. This pattern supports the view that optimisation efforts in alkali activation of mining tailings should prioritize process condition design over tailing selection alone, particularly in contexts where tailing type is dictated by the available mining operation.

### Activators

Table 3 presents the distribution of alkali activators used in each evaluated study. Most experiments used a combination of NaOH and sodium silicate, followed by NaOH alone and sodium silicate. The inclusion of soluble silicate increases the SiO_2_ content in the system, while the presence of hydroxide ensures the high alkalinity of the solution (Rocha et al. 2018).

**Table 3:** Types of activators found and the number of publications that used these activators to generate alkali-activated products from mining tailings

Activator type	Number of publications
NaOH and sodium silicate	18
NaOH	16
sodium silicate	16
KOH	3
NaOH, KOH, and sodium silicate	1
Phosphoric acid	1
Potassium aluminate and KOH	1
Potassium silicate and KOH	1
Sodium carbonate and sodium silicate	1
Sodium sulfate and sodium silicate	1

**Fig. 4:**
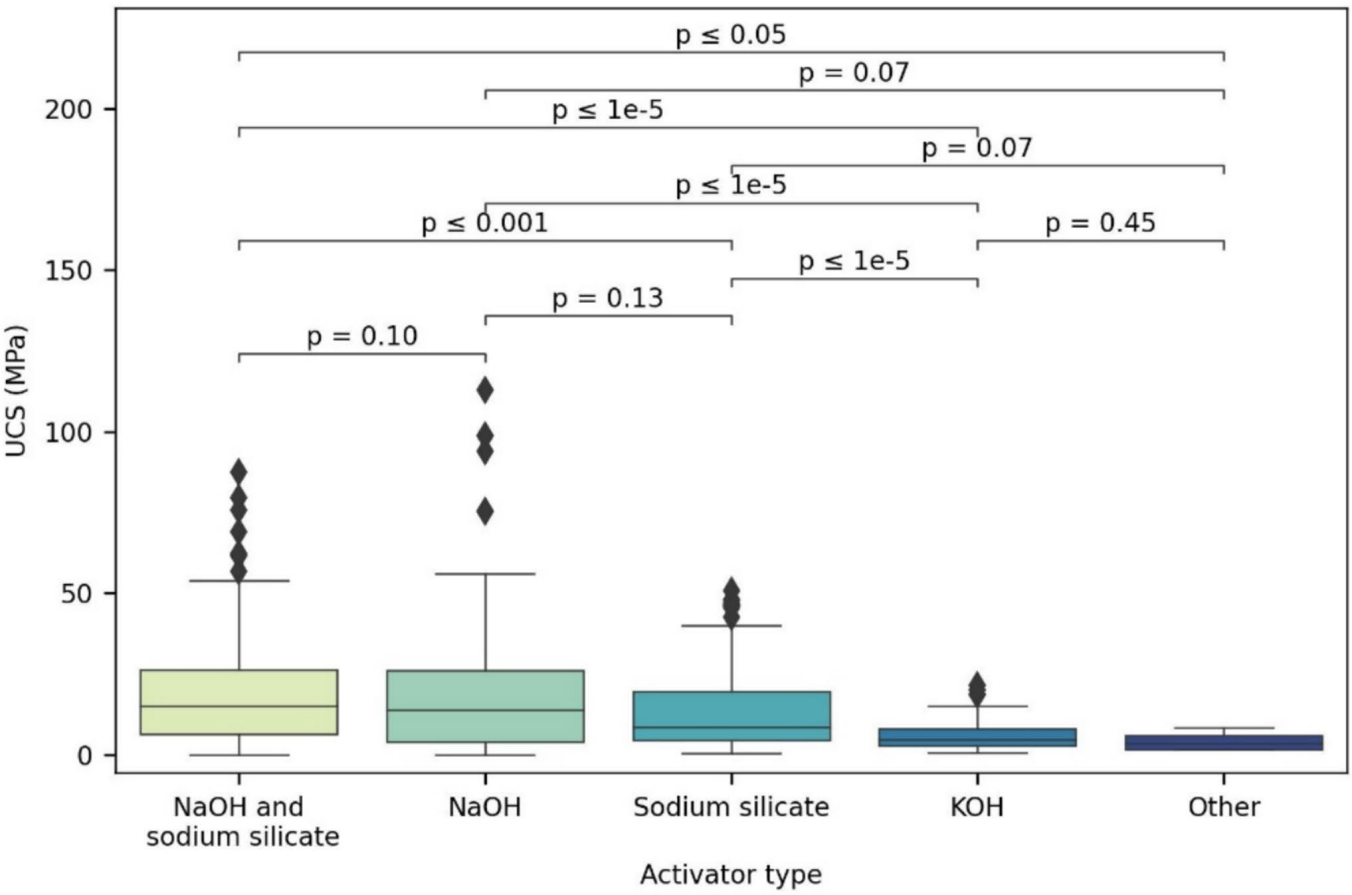
Boxplots of UCS values for experiments that used different types of alkali activators. The values above the boxes represent the *p*-values obtained via hypothesis testing

To identify significant differences between groups of experiments that used different types of activators concerning UCS values, *p*-values were calculated using the Mann–Whitney-Wilcoxon hypothesis test, as the data is not normally distributed. Figure 4 shows that there are statistically significant differences (*p* < 0.05) between the group using NaOH/sodium silicate and sodium silicate only, as well as significant differences between the former and KOH and others (the mixture of the two yielded a higher mean UCS). NaOH/sodium silicate did not show significant differences in UCS values when compared to NaOH.

Regarding the median UCS values for each group, the order from the highest median to the lowest is NaOH/sodium silicate, NaOH, sodium silicate, KOH, and others. Various studies that evaluated the mechanical response of alkali-activated products with different types of alkali activators indicated that the properties of these materials vary according to the type of activator used. According to Rocha et al. (2018), NaOH resulted in higher mechanical strength in the evaluated samples, while potassium silicate provided a uniform and dense morphology. Fernández-Jiménez and Palomo (2005) assessed the impact of three types of activators on the alkali activation of fly ash: NaOH, NaOH + sodium silicate, and NaOH + sodium carbonate. They found that the addition of sodium silicate to the NaOH solution increased the degree of condensation and, consequently, mechanical strength. On the other hand, the addition of carbonate ions was unsatisfactory due to the formation of sodium bicarbonate, which increased the acidity of the system and reduced reactivity between components. Zhang et al. (2012) recommend the use of activators with dissolved silicates instead of just hydroxide and carbonate because the resulting products tend to have higher strength and denser microstructures.

However, silicate-based activators are more chemically complex and may reduce crystal formation compared to hydroxide alone. Phoo-ngernkham et al. (2017) observed that using a mixture of sodium silicate and sodium hydroxide, or just sodium silicate, in alkali-activated materials made from fly ash and blast furnace slag resulted in higher compressive strength values. This increase was especially evident in mixtures with a significant amount of calcium. When it comes to mortars made from fly ash and Portland cement, mechanical properties were more satisfactory when a mixed solution was employed. In these cases, the silica present in the solution reacts rapidly with the calcium from the precursors, leading to an increase in mechanical strength. The presence of sodium hydroxide is crucial for the solubilization of silica and alumina, although these solubilizations and subsequent reactions are limited at room temperature.

#### Activator content

The amount of alkali activator in alkali-activated systems is a crucial factor that can significantly influence the properties of the resulting material. The concentration of the alkali activator can affect the reaction rate, product formation, mechanical strength, durability, and environmental stability of the system. However, the relationship between the amount of activator and system properties is not linear and may vary depending on other factors such as precursor composition, curing temperature, and time. As an example, Table 4 presents descriptive statistics for the dependent variable of strength (UCS), separated by the type of precursor used (100% tailings, 100% other precursor, and a combination of tailings and another co-precursor). It can be observed that the median strength values are higher for samples that used another type of precursor (commonly used ones such as fly ash and blast furnace slag), followed by the group that used a combination of precursors, and lastly, the group that used only tailings. This is strongly associated with the composition of these precursors, given their availability of essential elements for alkaline reactions (whether they are more amorphous or crystalline materials). This behavior justifies the need to evaluate the three groups separately to obtain clearer insights into the observed results.

**Table 4:** Descriptive statistics for the dependent variable UCS (MPa) grouped by the type of precursor used in the alkali-activated mixture

	***n***. samples	mean	std	min	25%	50%	75%	max
100% tailing	319	9.7	14.5	0.0	2.3	4.6	12.5	112.8
100% other precursor	87	23.3	19.5	0.2	7.1	21.0	33.9	87.4
Combination tailing/co-precursor	328	19.8	13.8	1.4	8.2	16.3	28.2	62.2

**Fig. 5:**
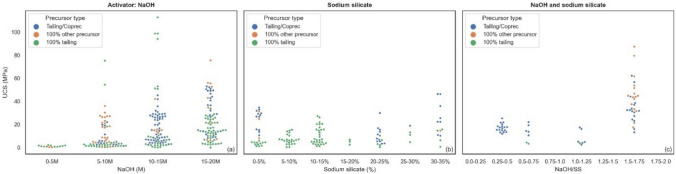
Distribution of UCS (MPa) for samples separated by precursor type and for **a** NaOH molarity, **b** % of sodium silicate, and **c** NaOH/SS ratio for activator mixtures

In this context, Fig. 5 presents the distribution of UCS (MPa) for samples separated by the type of precursor and the type of alkali activator used. It can be identified that, for NaOH activator (Fig. 5a), higher strengths are observed for higher concentrations (> 10 M). Additionally, it is noticed that samples composed of 100% tailings usually used concentrations in the range of 15–20 M, where higher UCS values were observed compared to cases that used 100% tailings but lower concentrations (achieving, in most cases, UCS values below 10 MPa). On the other hand, the higher concentration of samples using a combination of tailings and another type of co-precursor was constituted with molarities in the range of 10–15 M, obtaining UCS responses like the group of 100% tailings with higher NaOH concentration. When evaluating cases where another common type of precursor was used, there is a higher concentration of samples in the 5–10 M range, with results similar to the other two precursor types with higher activator concentrations. It is also important to consider that an excess of NaOH can lead to a decrease in compressive strength, as it may disturb the ideal Na/Al ratio. Furthermore, the decrease in strength with higher NaOH concentration may also be related to the formation of undesirable phases or the presence of unreacted NaOH in the matrix (Ren et al. 2015; Manjarrez and Zhang 2018).

Regarding sodium silicate (Fig. 5b), there is no clear trend of increased strength as the percentage of the activator in the mixture increases. Most samples were generated using a percentage of up to 15% SS. However, it can be observed that, for each concentration range, the highest strengths are observed for cases where a mixture of tailings with another type of precursor was used. This low observed relationship between the % of SS and the increase in compressive strength is influenced, in addition to experimental variables—curing time and temperature, for example—by the optimal Si/Al ratio (Wang et al. 2019). As observed by Wang et al. (2019), an excess of SS can disturb the optimal Si/Al ratio throughout the alkali activation reaction, leading to a decrease in compressive strength.

For cases where a mixture of activators was used (between NaOH and sodium silicate) (Fig. 5c), the highest UCS values were obtained with samples where the NaOH/SS ratio was greater than 1.5. Additionally, these samples are composed of mixtures of co-precursors (tailings and another co-precursor) or only an alternative precursor that is not tailings. Also, there was a lack of samples that tested the combination of these two activators in systems that used only tailings as a precursor.

Other types of activators (such as KOH and others) were not evaluated here due to a lack of sufficient samples for a more consistent analysis. These findings collectively indicate that the dominance of NaOH and sodium silicate in the literature is not merely a convention but reflects their demonstrated effectiveness across multiple system types. However, the near-absence of alternative activators (KOH, sodium carbonate, potassium silicate) in tailing-based systems represents a gap in the evidence base, leaving their potential largely untested under the specific conditions imposed by mining tailings as precursors.

#### Tailing as the sole precursor

**Fig. 6:**
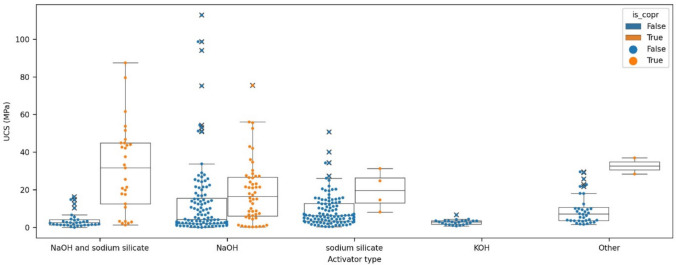
Boxplots of UCS values for samples that used 100% tailing in the mixture and samples that used 0% of tailings (100% of another precursor type)

Figure 6 presents the UCS values for two distinct groups: samples that used 100% tailing in the alkali-activated mixture and samples that used 100% of another commonly used co-precursor, such as metakaolin, fly ash, or GGBS, for example. A clear distinction in mechanical strength between these two groups can be identified (Tables 5 and 6). When analyzing samples with tailings only, it is found that experiments using NaOH and sodium silicate separately ended up observing more significant UCS values than in cases where a mixture of activators was used (Table 7).

**Table 5:** Mann-Whitney-Wilcoxon statistics and *p*-values for sets of samples separated by the type of activator used to identify differences between groups that used 100% tailing as a precursor or 100% of another type of precursor that is not tailing. The UCS variable was used as the dependent variable

Activator type	*U* statistic	*p*-value	Samples (100% tailing)	Samples (100% other precursor)
NaOH	3895.5	0.0026	52	116
NaOH and sodium silicate	850.5	1.60e-07	29	33
Sodium silicate	369.0	0.0253	4	111
Other	70.0	0.0285	2	36
KOH	-	-	0	23

**Table 6:** 75th percentile values for the UCS variable for each activator and precursor type

	NaOH	Sodium silicate	NaOH and sodium silicate	KOH	Other
100% tailing	15.52	12.77	4.09	3.44	10.65
100% other precursor	26.62	26.36	44.85		34.79

**Fig. 7:**
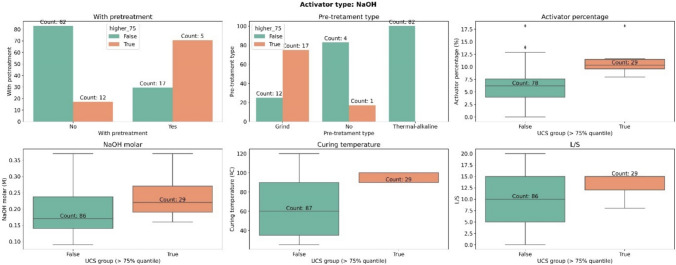
Distribution of variables that showed the greatest difference between groups of higher and lower UCS values (separated by the 75th percentile) for NaOH as the activator and only samples with 100% tailing as the precursor

Considering the 75th percentile values (Table 6) for cases where only tailing was used as a precursor, some variables were identified that showed greater separability for cases where UCS is higher than the quartile threshold or lower. For NaOH, it was observed that the highest UCS values used some form of pre-treatment, especially the grind method. Additionally, the percentage of activator used is higher for the group with the highest values for this variable, with, in most cases, molarities above 10 M. Regarding curing temperature, the higher resistance group was generated at curing temperatures above 90 °C. The L/S variable also showed higher values for this group (> 0.2) (Fig. 7).

**Fig. 8:**
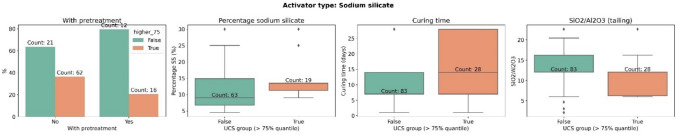
Distribution of variables that showed the greatest difference between groups of higher and lower UCS values (separated by the 75th percentile) for sodium silicate as the activator in samples with 100% tailing as the precursor

For samples that used sodium silicate, no significant difference was identified for the same variables mentioned for cases using NaOH as the activator. The main difference observed was for the SiO2/Al2O3 ratio of the precursor (tailing), where samples for the group above the 75th percentile for UCS represented lower values of the ratio when compared to the other group. For NaOH, this difference was not as apparent since the range of values studied for SiO2/Al2O3 was 4–9 for all cases (precisely the range of values observed for the optimal group of samples using SS). Variables such as the use of pre-treatment, the percentage of SS, and curing time and temperature did not show significant differences between the groups (Fig. 8). Regarding pre-treatment, when SS is used as an alkali activator, this pre-treatment may become less effective since the activator itself is a source of silicate that provides silicate ions in the solution. Therefore, the introduction of additional silica into the system by the activator itself can reduce the need for silicate provided by pre-treatment.

Samples that used a combination of activators with NaOH and sodium silicate with 100% tailings showed lower UCS values compared to the activators applied individually (Fig. 6 and Table 6). Thus, as the number of samples is low, it is not possible to obtain more reliable insights for samples with these characteristics, requiring the development of more experimental studies involving these conditions. This finding has a practical implication: for systems where only tailings are available as a precursor, the use of individual activators, particularly NaOH at concentrations above 10 M combined with curing temperatures above 90 °C, appears to be a more reliable strategy than activator combinations, at least based on the evidence currently available in the literature.

#### Tailings in combination with other precursors

**Fig. 9:**
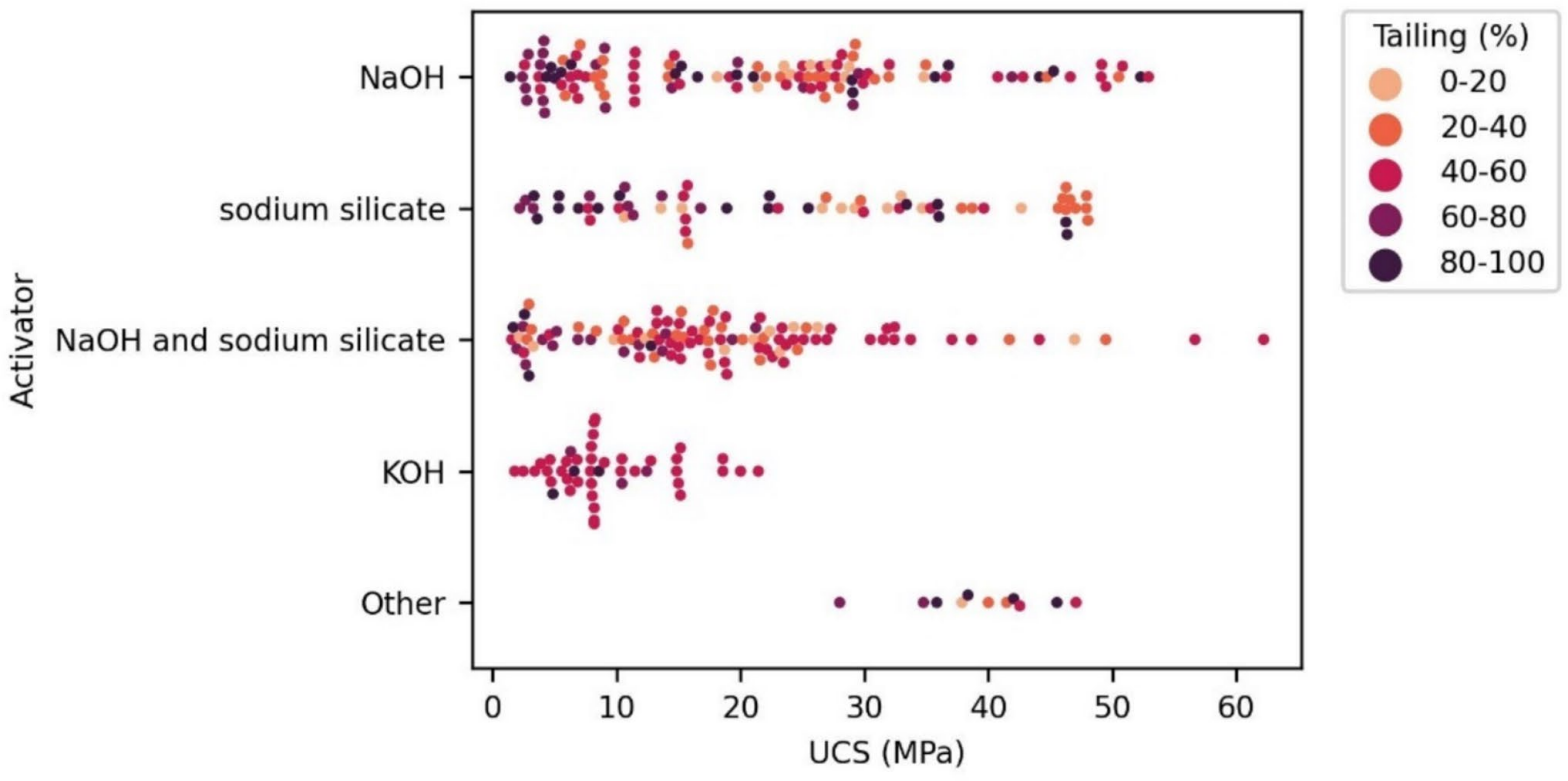
Response of mechanical strength for samples with a combination of tailing and another co-precursor grouped by the type of alkali activator used

Figure 9 presents the distribution of samples that used combinations of tailings and co-precursors in the alkali-activated system regarding their performance response, along with information on the percentage of tailing used in the preparation of each sample. It can be identified that for combination cases, studies typically use a median value of 50% tailing/co-precursor. Additionally, there is also a trend of lower UCS values for samples that used at least more than 60% of tailing in the mixture, which corroborates with the results presented in previous sections where the lower UCS values were observed for the group that used 100% tailing.

**Fig. 10:**
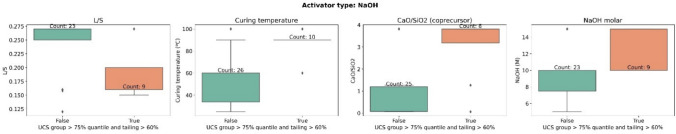
Variables that showed the greatest difference between groups of higher mechanical strength (75th percentile) and used more than 60% of tailing and NaOH as the activator

Specifically, for NaOH, samples with high mechanical strength were observed even with a high content of tailing in the mixture (> 60%). The characteristics of these samples, when compared to samples with the same tailing content, are represented by lower values of L/S (0.14–0.18), high curing temperatures (90 °C), co-precursors with a higher CaO/SiO2 ratio, and higher NaOH values (> 10 M) (Fig. 10).

### Influence of pre-treatment

In the alkali activation process, pre-treatment techniques can be used to improve the reactivity of precursors, increase process efficiency, and ultimately enhance the quality of the final product (Kuranchie et al. 2016; Wang et al. 2019; Wu et al. 2020). Therefore, it is an important variable to be evaluated for consideration in the process. Pre-treatment can involve various techniques such as grinding, heating, or a combination of both.

Among the evaluated studies, 28 did not apply any form of pre-treatment to the precursors (rejects), while 22 implemented some form of pre-treatment. A more detailed analysis of these 22 articles revealed a variety of pre-treatment methods used. Grinding was the most common method, employed in 12 studies. Heating was used in 2 studies, while a combination of heating and grinding was adopted in 7 studies. Only one study used the thermal-alkali method.

**Fig. 11:**
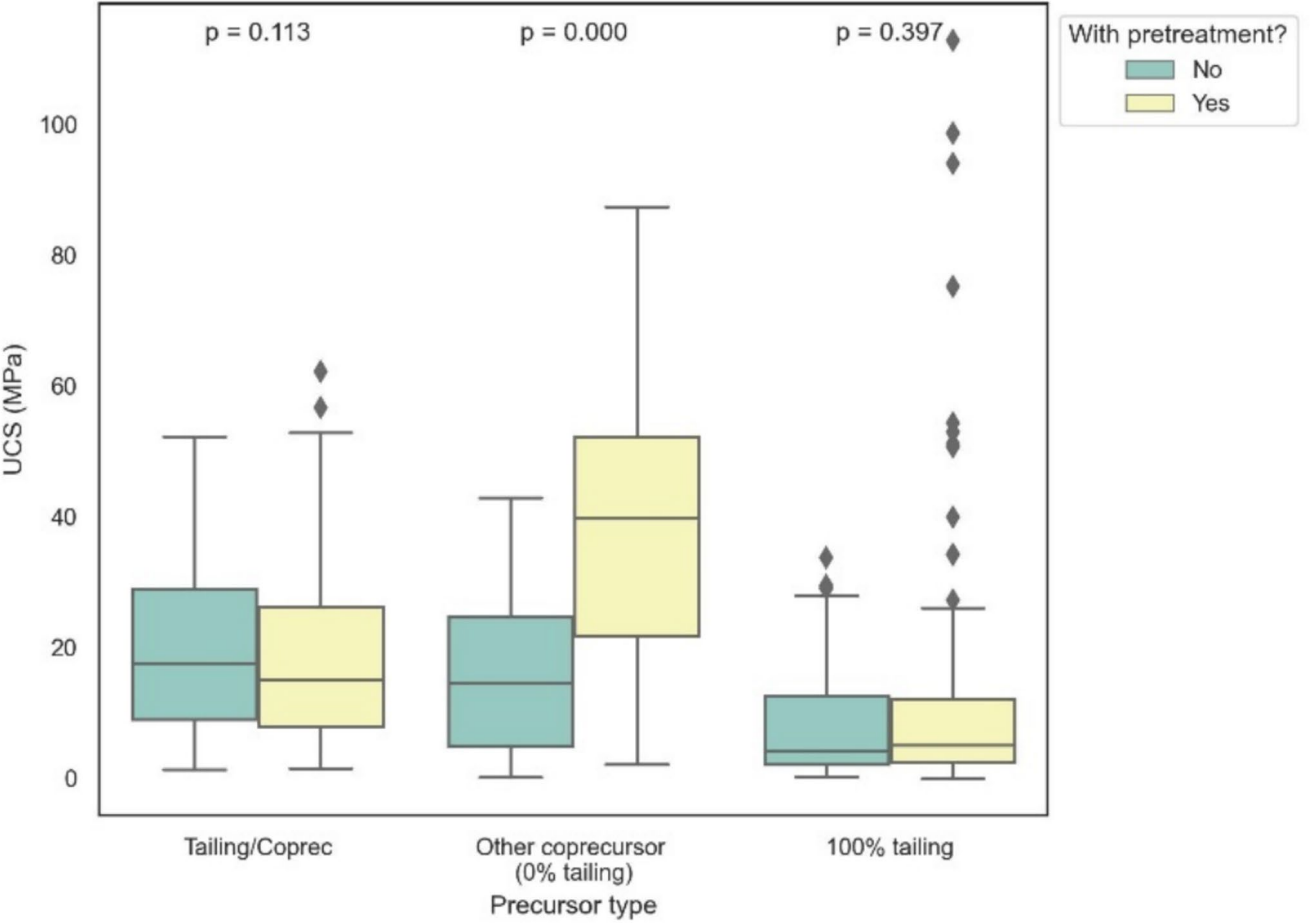
Distribution of UCS values for samples that used or did not use any pre-treatment method grouped by precursor type. The *p*-values presented at the top of the graph indicate the significance obtained by the Mann-Whitney test between the groups with/without pre-treatment

From Fig. 11, it can be observed that the group that shows a statistically significant difference between samples with and without pre-treatment is the one where 100% of another type of precursor other than tailing was used (fly ash, GGBFS, among others commonly used in alkali activation). This may be related to the fact that, as ordinary precursors already have suitable characteristics for incorporation into the process, the use of pre-treatment can enhance the effect more intensely. On the other hand, when another type of precursor with different characteristics is used, such as tailings, which have highly crystalline structures, for example, pre-treatment may not be very effective. The low reactivity of materials, even after grinding, can lead to the existence of unreacted residues and an increase in the number and size of large voids, resulting in a decrease in compressive strength (Zhao et al. 2021).

**Fig. 12:**
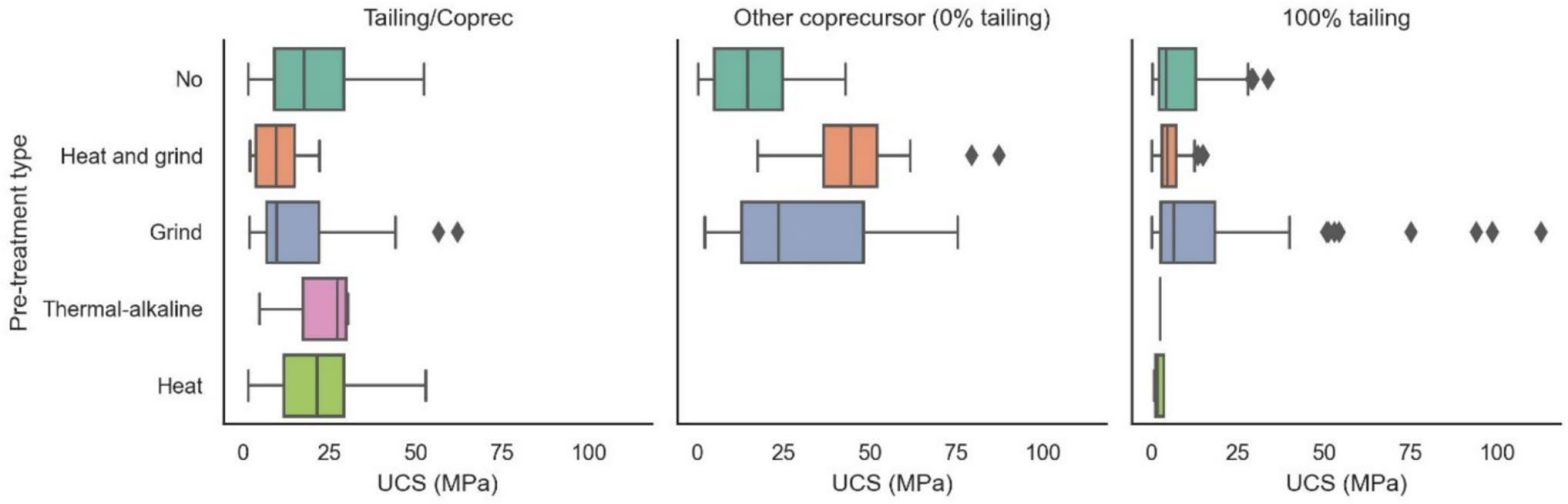
Distribution of UCS values for samples with different types of pre-treatments, grouped by the type of precursor used

Few samples exceed the maximum strength value for the group without pre-treatment when considering groups that used a portion of tailing in the system (Fig. 12). For the case where 100% tailing was used as a precursor, the main difference between the group of samples that obtained higher strength than the maximum strength value of the group without pre-treatment (33.76 MPa) lies in the temperature variable, where values above 80 °C were applied. The maximum strength observed for this group (112.80 MPa) was obtained with a curing temperature of 100 °C, NaOH with 12 M as alkali activator, iron ore tailing as the precursor, and 3 h of grinding as the pre-treatment method (do Carmo e Silva Defáveri et al. 2019). For samples where a combination of tailing and another co-precursor occurred, only 3 samples exceeded the maximum strength value of the group without pre-treatment (> 52.29 MPa). These samples also have characteristics such as high curing temperatures (> 80 °C), and a curing time greater than the group with lower UCS values (> 20 days). The tailings in this group are phosphate (Moukannaa et al. 2018) and copper Ahmari et al. (2015), the first characterized by a high CaO/SiO2 ratio (1.5).

### Influence of co-precursor

The addition of co-precursors in an alkali-activated system can be useful and necessary, especially when dealing with precursors from waste or tailings that do not possess all the necessary characteristics to obtain a higher-strength alkali-activated product. Firstly, waste materials may have low chemical reactivity and low mineral content, limiting their effectiveness as precursors (Borges et al. 2019). By incorporating other co-precursors such as fly ash or metakaolin, known for their high pozzolanic activity, the overall reactivity and strength of the resulting materials can be improved (Liu et al. 2021). Additionally, the combination of different co-precursors can lead to the formation of a more diverse range of reaction products, resulting in improved mechanical properties and durability (Perumal et al. 2020). Furthermore, the availability and cost-effectiveness of co-precursors can vary depending on the region and specific mining operations, necessitating the exploration of alternative precursor sources (Obenaus-Emler et al. 2020).

**Fig. 13:**
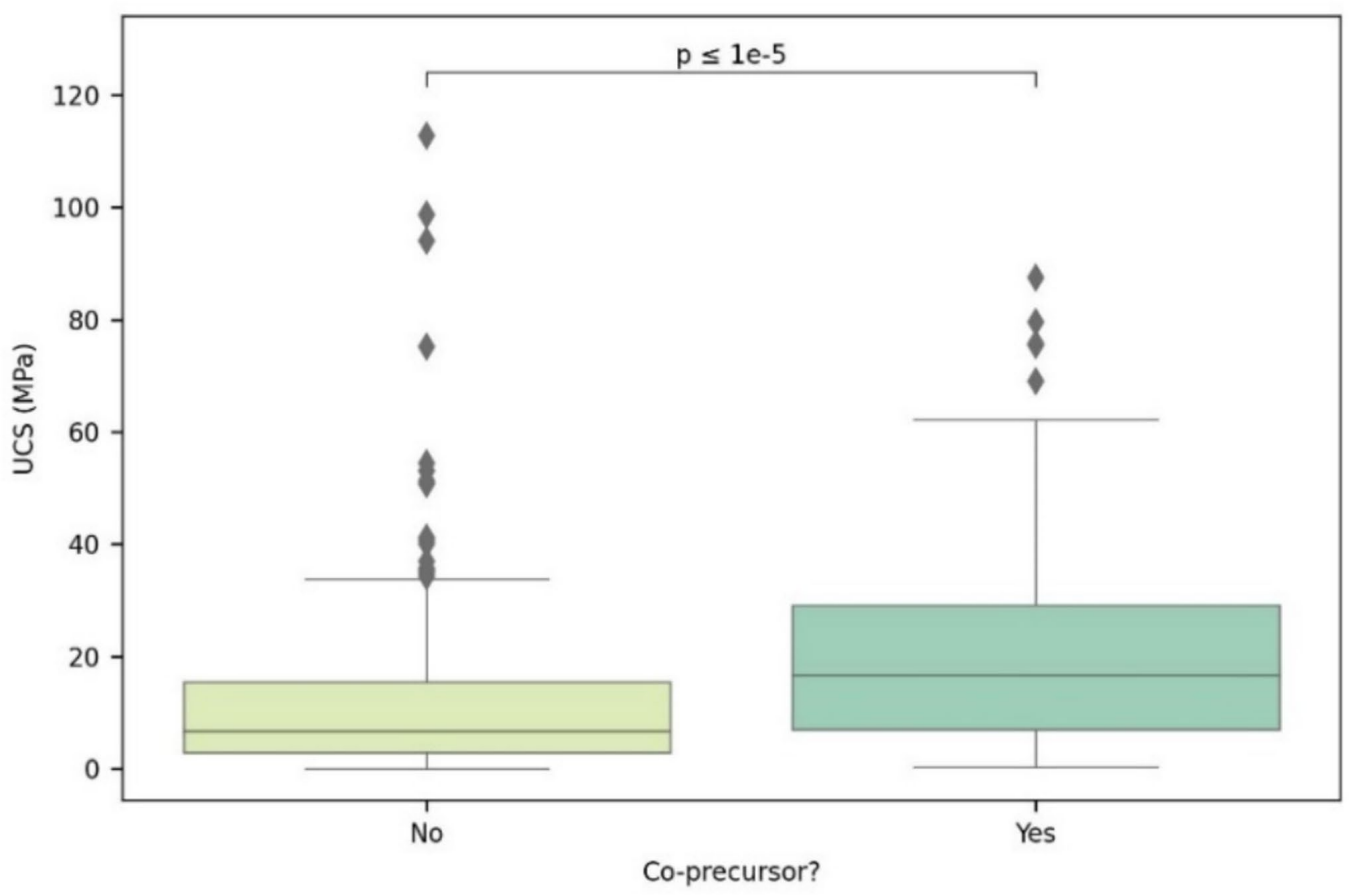
Distribution of UCS values for the group that used some type of co-precursor in addition to tailing, and the group that used only tailing. *p*-value is given by the Mann-Whitney statistical test

Based on the analyzed literature, 39 studies did not employ a co-precursor at any stage of the experiment, while 28 opted to use one or a combination of these at some point in the experimental phase. It is important to note that a single study may have both used and not used a co-precursor, which is why the total sum of articles is higher in this context. Statistical analysis reveals a significant difference between alkali-activated products that used a co-precursor and those that did not (Fig. 13). This supports the results presented in the previous sections, where higher UCS values were observed in cases where there was at least a combination of tailings and another type of co-precursor. However, no significant statistical difference was observed for cases where two or more co-precursors were applied in addition to the tailings.

**Fig. 14:**
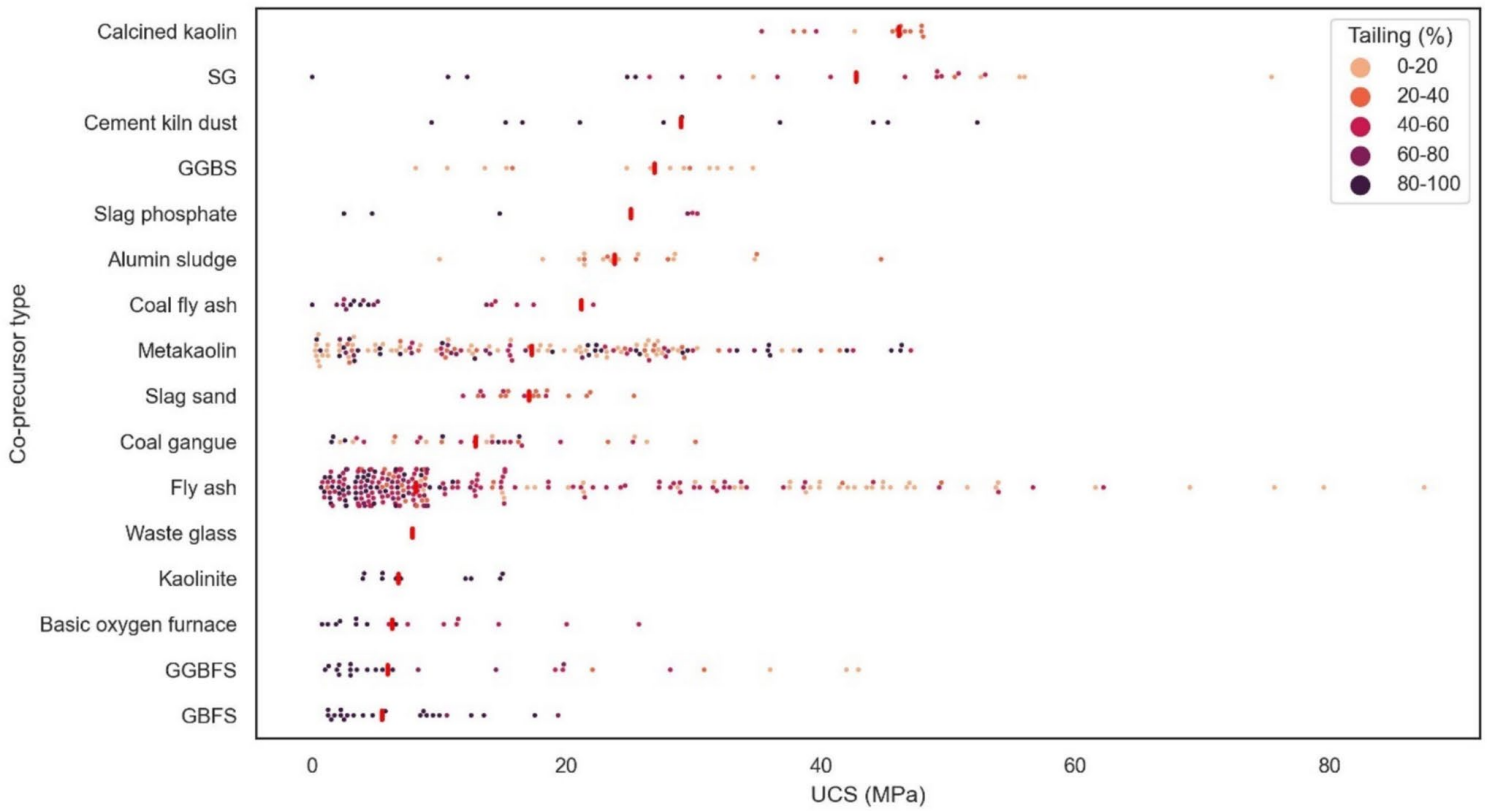
Types of co-precursors used in the reviewed studies and distribution of UCS values for each sample using a co-precursor. The red bar indicates the median UCS value. The colors represent the percentage of tailings present in each sample in relation to the added co-precursor

Figure 14 presents the distribution of UCS values for each type of co-precursor used, along with information on the percentage of tailings present in that mixture. The most used co-precursors in alkali activation studies with tailings were fly ash and metakaolin. Furthermore, as observed in Fig. 3, the highest UCS values, for all types of co-precursors, are associated with lower percentages of tailings in the mixture. Ordinary precursors, such as fly ash and metakaolin, have higher reactivity and finer particle size compared to tailings, allowing more silica and alumina species to dissolve, and be incorporated into the reaction (Manjarrez and Zhang 2018). This leads to the formation of a stronger matrix with higher bonding strength between particles (Zhang et al. 2011). Moreover, a higher percentage of co-precursor results in a lower Si/Al ratio, which is closer to the ideal Si/Al ratio for geopolymerization (Longos et al. 2020). On the other hand, tailings exhibit lower reactivity and larger particle sizes, which can limit the alkali activation process and result in lower UCS values (Niu et al. 2020).

The ideal percentage of co-precursor to be used with tailings is challenging to determine due to various factors. Firstly, the composition of tailings can vary significantly depending on the type of mining operation and the specific ore being processed. This variability makes it difficult to establish a one-size-fits-all approach for co-precursor selection. Additionally, interactions between co-precursors and tailings can be complex and not fully understood, making it difficult to predict the outcomes of different combinations and percentages. Nevertheless, the statistically significant difference in UCS observed between systems with and without co-precursors (Fig. 13) provides clear evidence that co-precursor addition is one of the most consistently effective levers available to improve mechanical performance in alkali-activated tailings systems, more reliable, based on the compiled data, than increasing activator concentration or curing temperature alone.

### Curing temperature

The curing temperature is a crucial aspect of the alkali activation process. Curing temperature can significantly influence the reaction rate and, consequently, the properties of the final product. An appropriate curing temperature can ensure a complete reaction, resulting in a material with higher strength and durability. However, excessively high curing temperatures can lead to overly rapid reactions, causing the formation of micro-fissures and reducing the quality of the final product. Therefore, the choice of curing temperature is a delicate balance that must be carefully managed to optimize the alkali activation process.

In the evaluated studies, temperatures used in the experiments ranged from 20 to 120 °C. Table 7 presents the minimum, median, and maximum values for each precursor group. The median tested temperature for all groups was 60 °C.

**Table 7:** Curing temperatures used in experiments from evaluated studies, grouped by precursor type

Precursor type	*N*. samples	Min (°C)	Median (°C)	Max (°C)
100% tailing	315	20	60	120
100% other precursor	82	20	60	105
Tailing/co-precursor	306	20	60	105

**Fig. 15:**
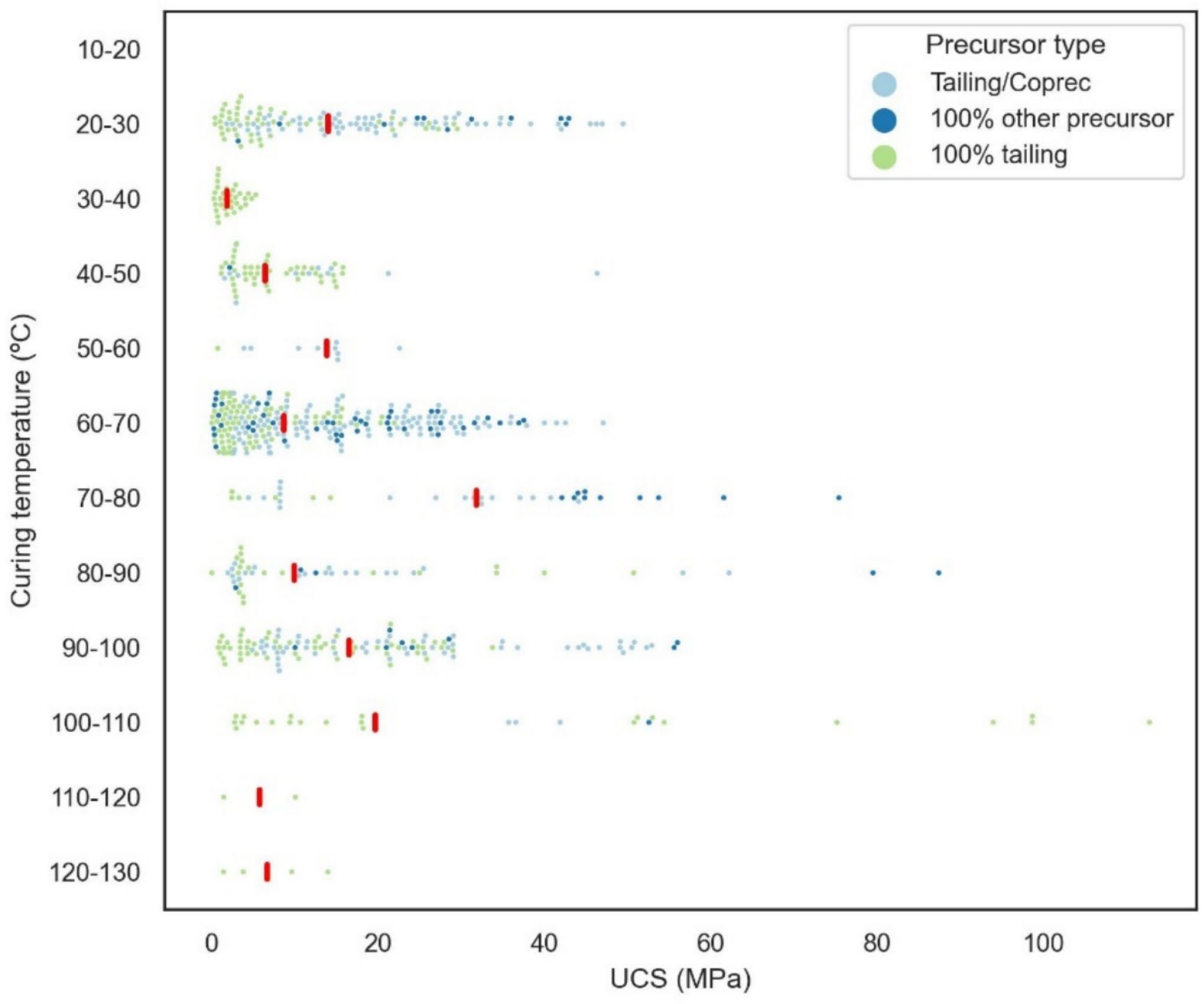
Distribution of UCS (MPa) values for each curing temperature range. The red bar represents the median UCS values. Colors indicate the type of precursor used

When evaluating only the group that used 100% tailing as a precursor, it is observed that higher strengths were obtained at higher curing temperatures, above 80 °C (Fig. 15). Higher temperatures can improve the solubility of alumina species and provide more energy for molecular mobility, promoting the development of a denser structure. However, for cases that used tailing with a co-precursor, higher strengths are already observable even at lower temperatures (Zhang et al. 2011; Liu et al. 2021).

Although an increase in strength at high temperatures can be observed, this is not always true. In Fig. 15, it can be observed that even at high temperatures, low strengths were obtained. Higher curing temperatures can lead to a decrease in compressive strength due to pore production, resulting in low density and resistance (Wang et al. 2019). Additionally, the loss of physically and chemically bound water at higher temperatures can result in the presence of large macropores or voids, which can decrease strength (Koshy et al. 2019). Moreover, an overdose of alkali activator in this temperature range can weaken the material’s strength due to carbonate formation (Zhao et al. 2021). Finally, the simultaneous increase of certain factors, such as activator concentration, type, and curing time, can have a negative impact on density and strength (Moukannaa et al. 2018).

Importantly, the dependency of tailing-only systems on high curing temperatures has a direct practical implication: it increases energy costs and limits the applicability of these systems in field conditions. The fact that tailing/co-precursor combinations can achieve competitive UCS at ambient or moderate temperatures therefore represents not only a mechanical advantage but also an operational and economic one.

### Curing time

Curing time can directly influence the effectiveness of the alkali activation reaction and, consequently, the characteristics of the final product. Adequate curing time can ensure a complete reaction, resulting in a material with higher strength and durability. However, excessively long curing times can lead to an overly slow reaction, compromising the quality of the final product. Therefore, determining the curing time is a delicate balance that must be carefully managed to optimize the alkali activation process.

**Fig. 16:**
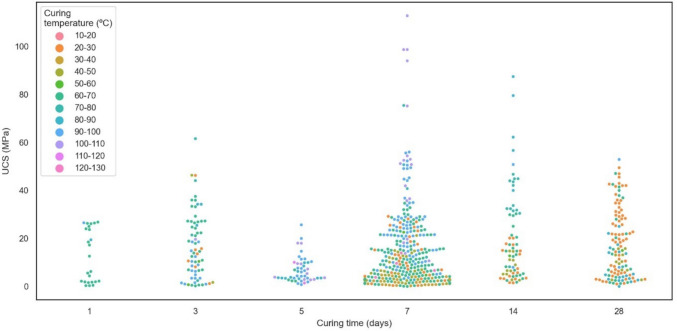
Distribution of UCS (MPa) values by curing time, in days, and curing temperature

In the evaluated studies, curing times ranged from 1 to 28 days, with most samples evaluated at a standard time of 7 days (Fig. 16). An interesting point is that, for most samples that used a lower curing temperature (20–30 °C), the curing time in the evaluation was 28 days (Kiventerä et al. 2016, 2018; Chen et al. 2020).

However, curing time does not seem to consistently influence the final strength value (Fig. 16). For example, Zhang et al. (2011) observed that a significant portion of the ultimate compressive strength was obtained in 2 days, and samples reached their ultimate compressive strength in 7 days. After 7 days, the final compressive strength essentially remained unchanged.

### L/S

The liquid-to-solid ratio in the alkali activation process refers to the proportion between the amount of liquid (usually an alkaline solution) and the amount of solid (usually a silicate material) in the mixture. This ratio is an important parameter that influences the properties of the product. An appropriate liquid-to-solid ratio is necessary to ensure that the solid material is fully activated by the alkaline solution, resulting in a product with the desired properties. Adjusting this ratio allows control over the viscosity of the mixture, the reaction rate, product strength, and other important properties. Therefore, the liquid-to-solid ratio is a critical factor in the alkali activation process.

A very high liquid-to-solid ratio can result in a too-fluid mixture, leading to segregation of solid components. On the other hand, a very low liquid-to-solid ratio can result in a too-thick mixture, making it difficult to properly mix the components. Therefore, optimizing the liquid-to-solid ratio is essential to ensure the effectiveness of the alkali activation process.

**Fig. 17:**
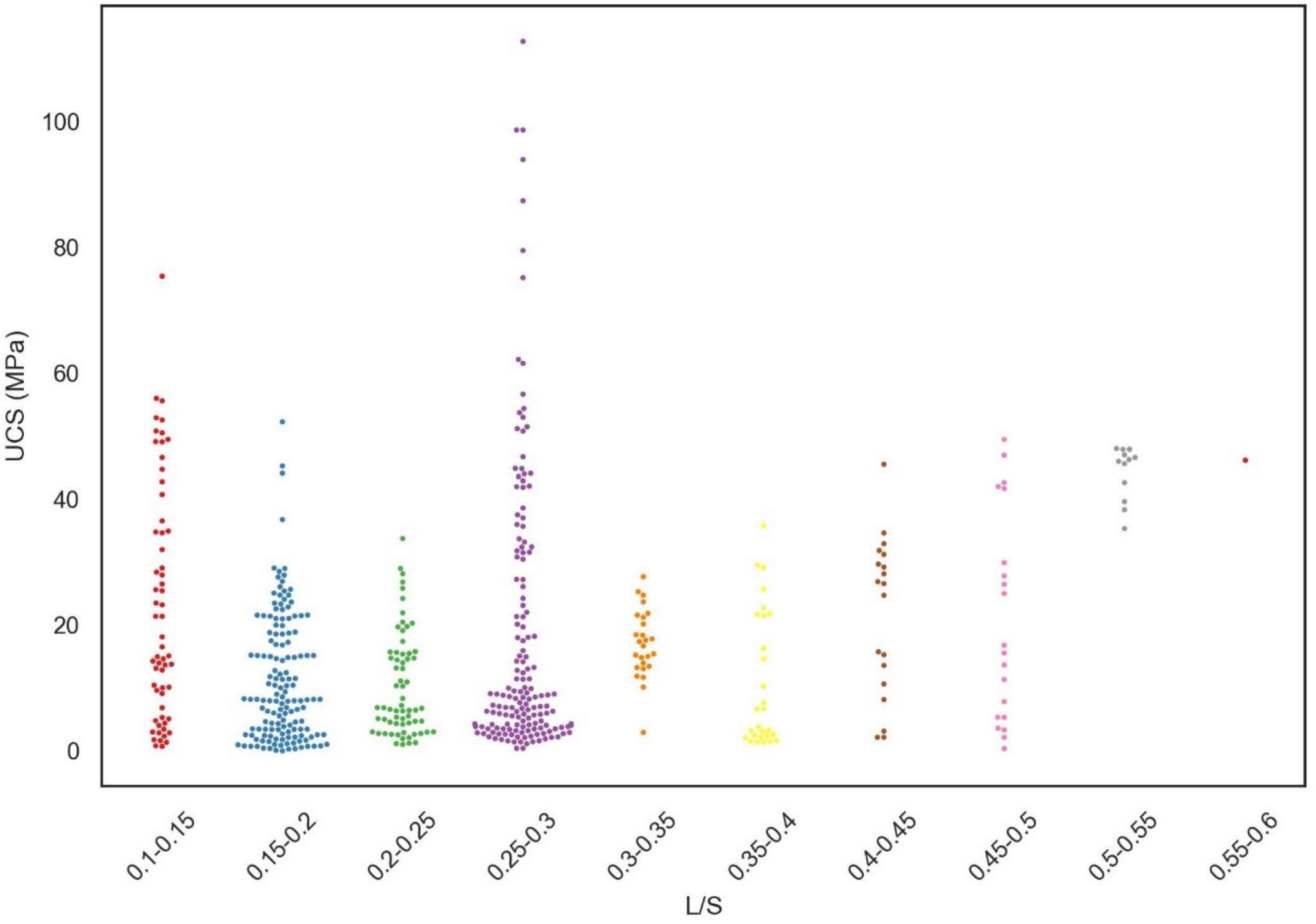
Distribution of UCS based on different ranges of L/S variable

In the evaluated literature, the L/S ratio varied from 0.1 to 0.6 in experimental programs, with a median value of 0.26 (Fig. 17). Like other variables, the optimal L/S value depends on the characteristics of the precursors and activators used. For example, in a system with tailings and calcined kaolin and sodium silicate as an activator, Cao et al. (2013) tested different L/S values and found the best value to be 0.6. Low ratios resulted in handling difficulties, while high ratios weakened the material. A ratio of 0.6 achieved a balance, offering optimal strength and workability. With the 0.6 ratio, the material reached its maximum compressive strength (~ 45 MPa). On the other hand, Tian et al. (2020b) tested L/S values in the range of 0.13 and 0.17 in an alkali-activated system with copper mining tailings and fly ash as precursors, a mixture of sodium silicate and sodium hydroxide, and calcium oxide as activators. A decrease in compressive strength was observed when the L/S ratio was 0.17. The highest compressive strength of 40.9 MPa was achieved with an L/S ratio of 0.15. The contrasting optimal L/S values reported across studies (0.15 to 0.60) reflect the strong dependency of this parameter on the specific combination of precursor and activator used, confirming that no universal optimal ratio can be established from the aggregated literature. Nevertheless, the data compiled here suggest that most systems achieving UCS values above the dataset median operated within an L/S range of 0.15–0.35, which may serve as a practical starting reference for experimental programs involving untested tailing types.

## Final remarks

The analysis derived from different experiments provided an overall overview of the experimental conditions studied and the achieved strength results. Additionally, it was possible to identify experimental characteristics that led to higher strengths. The main considerations are as follows:

- Copper tailings were the most frequently studied precursor in the evaluated literature, followed by gold, iron ore, and red mud tailings. Among all tailing types, iron ore and phosphate tailings exhibited the highest median UCS values, a result associated not with their intrinsic reactivity alone, but with the experimental conditions predominant in the studies that used them, particularly the use of co-precursors, high activator concentrations, and elevated curing temperatures
- The type of precursor used affects the mechanical strength result: a median of 4.6 MPa was observed for samples with 100% tailing as a precursor, with 75% of the samples below 12.5 MPa. For combinations of tailing/co-precursor, the median is higher, obtaining median values of 21 MPa, and a 75% quartile of 33.9 MPa
- NaOH and sodium silicate are still the most studied activators. For NaOH and samples with 100% tailings, the highest UCS values were obtained with molarities above 10 M and high curing temperatures
- The addition of co-precursors in addition to tailings helps increase mechanical strength. The most studied are fly ash and metakaolin
- No statistically significant difference was observed in the use of pre-treatment for evaluated tailing samples
- Curing temperatures above 80 °C resulted in higher strength values for samples that used only tailings. It is possible to obtain interesting strength values with lower temperatures if a common co-precursor is used
- Curing time above 3 days does not seem to generate a significant difference in mechanical response for the evaluated dataset.

Considering the variables used in the collected dataset and the obtained results, and considering the application in real-world and large-scale cases, it is necessary to seek milder conditions regarding the concentration of activators and temperature to avoid both environmental and financial high costs. This also includes the exploration of alternative activators and co-precursors. It is acknowledged that the temporal scope of the literature search (1991–2021) represents a limitation of the present study. As this work was developed within the framework of a doctoral thesis, the search protocol and temporal boundaries were defined and fixed prior to data collection and statistical analysis; studies published after 2021 were therefore not included. Nevertheless, the experimental conditions and activator systems identified as predominant in the compiled dataset, NaOH, sodium silicate, and their combinations, continue to represent the mainstream approaches in the field, and the cross-study statistical patterns identified here are unlikely to be reversed by more recent publications, though they may be further refined. Future work should update this dataset to incorporate recent literature and assess whether the patterns identified remain consistent as the field matures.

## Data Availability

The datasets used and/or analyzed during the current study are available from the corresponding author on reasonable request.
